# Rainfed assessment of foxtail millet (*Setaria italica* L. beauv) germplasms through genotyping and principal component analysis

**DOI:** 10.3389/fpls.2023.1017652

**Published:** 2023-03-08

**Authors:** Divya Singh, Kapil Lawrence, Shailesh Marker, Indranil Bhattacharjee, Reena Lawrence, Ravish Choudhary, Sezai Ercisli, Rohini Karunakaran

**Affiliations:** ^1^Department of Biochemistry and Biochemical Engineering, Jacob Institute of Biotechnology and Bioengineering, Sam Higginbottom University of Agriculture, Technology and Sciences, Prayagraj, Uttar Pradesh, India; ^2^Department of Genetics and Plant Breeding, Sam Higginbottom University of Agriculture, Technology and Sciences, Prayagraj, Uttar Pradesh, India; ^3^Department of Chemistry, Sam Higginbottom University of Agriculture, Technology and Sciences, Prayagraj, Uttar Pradesh, India; ^4^Division of Seed Science and Technology, ICAR-Indian Agricultural Research Institute, New Delhi, India; ^5^Department of Horticulture, Faculty of Agriculture, Ataturk University, Erzurum, Türkiye; ^6^Unit of Biochemistry, Faculty of Medicine, Semeling, Kedah, Malaysia; ^7^Department of Computational Biology, Institute of Bioinformatics, Saveetha School of Engineering (SSE), SIMATS, Thandalam, Chennai, Tamil Nadu, India; ^8^Centre of Excellence for Biomaterials Science, AIMST University, Semeling, Bedong, Malaysia

**Keywords:** genotypic coefficient of variation (GCV), phenotypic coefficient of variation (PCV), genotypic path, phenotypic path, principal component analysis

## Abstract

**Introduction:**

Foxtail millet (*Setaria italica* L. beauv) is an important crop in underdeveloped countries; however, yield levels are low. The use of varied germplasm in a breeding approach is critical for increasing productivity. Foxtail millet can be cultivated effectively in a wide range of environmental circumstances but it is best suited to hot and dry climates.

**Methods:**

In the current study, multivariant traits were used to define 50 genotypes in the first year and 10 genotypes in the second year. The phenotypic correlations among all traits in the entire germplasm were assessed, and the data acquired for all quantitative characters were subjected to analysis of variance for augmented block design. Furthermore, WINDOWS STAT statistical software was used to carry out a principal component analysis (PCA). The presence of substantial variations in most symptoms was shown by analysis of variance.

**Results:**

Genotypic coefficient of variation (GCV) projections for grain yields were the highest, followed by panicle lengths and biological yields. Plant height and leaf length had the highest PCV estimates, followed by leaf width. Low GCV and phenotypic coefficient of variation (PCV) were measured as leaf length and 50% flowering in days. According to the PCV study, direct selection based on characters, panicle weight, test weight, and straw weight had a high and positive effect on grain yield per plant in both the rainy and summer seasons, indicating the true relationship between these characters and grain yield per plant, which aids indirect selection for these traits and thus improves grain yield per plant. Variability in foxtail millet germplasm enables plant breeders to effectively select appropriate donor lines for foxtail millet genetic improvement.

**Discussion:**

Based on the average performance of genotypes considered superior in terms of grain yield components under Prayagraj agroclimatic conditions, the best five genotypes were: Kangni-7 (GS62), Kangni-1 (G5-14), Kangni-6 (GS-55), Kangni-5 (GS-389), and Kangni-4 (GS-368).

## 1 Introduction

Plant genetic resources (PGR) are the backbone of the agricultural system, playing a positive and distinguishing role in the development of new cultivars from the past to the present, including the restructuring of existing ones (Sapkota et al., 2016). Genes for such traits are typically available in wild animals and landraces, allowing for the development of genotypes that can endure biotic and abiotic pressures. The current study concentrated on the genetic diversity of wild crop relatives. Genetic diversity, endangered plant species, species diversity, ecosystem stability, global floristic diversity in food plants, genetic resources in India, wild collections of major crops, plant genetic resources vis-à-vis crop breeding emphasis, and conservation of plant genetic resources are among the information needed to develop a breeding plan for sustainable agriculture: foxtail millet is a C4 crop that is diploid (2n = 18) (Mohammadi and Prasanna, 2003; Aghaee-Sarbarzeh and Amini, 2012). Foxtail millet cultivation is currently restricted to a few pockets, and in some locations it has been replaced by other crops that require irrigation. Its high nutritional value, combined with its low water requirement, makes it a climate-resilient crop appropriate for production in dryland agricultural systems. It has a tiny genome, and its use as a model crop for bioenergy has generated much interest, with more groups working on it than ever before. This troop’s floral morphology and flowering behavior make it challenging to establish crosses between the desired parents. As a result, we have seen several published studies on creating strategies for crossing in foxtail millet to date. The experiment addresses floral biology, crossing procedures, and the generation of cytoplasmic male sterile (CMS) lines (Bhat et al., 2018).

The main component of foxtail millet grain is starch. Aside from grain, protein and fats are found in significant proportions. There are also some free sugar and non-starchy carbohydrates (CSE, 2007). Starch is widely used as a raw material in a variety of sectors, including textile, food, pharmaceutical, and paper. Native starch has relatively few industrial applications. Physical, chemical, or enzymatic processes can be used to create modified starches with specified qualities for a variety of uses (Kim et al., 2010). Owing to the rapid expansion of foxtail millet improvement in recent decades, as in other crops, foxtail millet landraces have been replaced by current cultivars, resulting in a significant loss of genetic diversity. As a result, established techniques of maintaining and multiplying foxtail millet landraces must be reconsidered. This could give germplasm conservationists and breeders some insight into the domestication, evolution, selection, and preservation of the world’s oldest cereal crop (Ahmed et al., 2013). Foxtail millet is a promising source of micronutrients and protein compared with other cereals. Foxtail millet grain is (per 100 g) rich in protein (12.3%), iron (2.8 mg), and calcium (31 mg) compared with rice (7.9% protein and 1.8 mg iron) according to the Millet Network of India (MINI). Additionally, they contain a high quantity of beta-carotene and have a higher proportion of non-starchy polysaccharides and dietary fiber. Foxtail millet releases sugars very slowly and thus has a low glycemic index (GI) and hence can be used in a therapeutic diet but its potential role as low GI food has remained unrealized and unexploited. The low glycemic index diet has been shown to reduce blood glucose levels (Ahmed et al., 2013).

For selecting a new variety, GPB hybridization is one of the most efficient methods at present, with the ultimate goal of selecting a new variety (Islam, 2004). Appropriate parental line selection is the most important aspect of a dry lab experiment to improve the genetic recombination of potential breeds (Verma et al., 2018; Singh et al., 2019). Additionally, a vast number of morphologically documented germplasm studies are needed to determine the differences between all germplasm populations and their breeding potential. Breeders assessed a huge number of germplasm varieties, some of which may or may not have enough discriminatory power for germplasm selection, characterization, assessment, and management (Maji and Shaibu, 2012). If this is the case, then principal component analysis (PCA) can be used to determine parentage and reduce duplication in experimental data sets in which morphological and physiological variation occurs on a regular basis in GPB sciences (Singh and Verma, 2020). PCA is a multivariate statistical methodology that seeks to simplify and analyze the relationships between a large number of variants in terms of a relatively small number of variables or components while retaining all crucial information from the original genotype data set. (Amy and Pritts, 1991; Adams, 1995). However, these genotypes of foxtail millets have not been systemically determined so far; therefore, the current investigation provides a detailed overview of the rainfed assessment of *S. italica* genotypes through genotyping and principal component analysis.

## 2 Materials and methods

The experimental material consisted of accessions from 2018 and 2019, including 50 germplasm accessions of foxtail millet from 2018 and 10 from 2019. These 50 germplasm accessions were collected from ICRISAT and NBPGR, New Delhi during Kharif 2018. For evaluation and characterization, these 50 germplasm accessions and three check varieties were grown in a randomized complete block design (RBD) at the Field Experimental Centre, Department of Genetics and Plant Biotechnology, SHUATS, Prayagraj, India. The selection of the 10 best genotypes from 2019 was based on the yield of 50 foxtail millet germplasm accessions. The characterization site, Naini Prayagraj, is located at 13° 05' N latitude and 77° 34' E longitude. The Centre is 924 m above mean sea level. The annual rainfall ranges from 528 to 1374.4 mm with a mean of 915.8 mm. The germplasm accession was divided into three blocks, each consisting of 46 accessions and four check varieties (ISE375, ISE1468, ISE132, and ISE376). Each accession was grown in a single row 3 m in length and spaced 30 cm apart, and plant-to-plant spacing within the row was 10 cm. After 15 days, the crop was supplied with the recommended dose of fertilizer (10 kg N and 20 kg P-05 ha as a basal dose and 10 kg N at the time of earthing up). Irrigation was not provided and crops only received rainwater and were protected from weeds, pests, and diseases. For all characters, except days to emergence and days to maturity, observations were made for five randomly selected plants in each entry of each replication. The phenotypic correlation coefficients were obtained using the formula proposed by Vinsonias (2018).

The phenotypic correlations of all traits in the complete germplasm were estimated, and numerous significant correlations were found. Data for all quantitative characters were collected and subjected to analysis of variance for augmented block design using the method described by Kempthorne (1957). PCA was calculated for 15 quantitative traits to examine the relative value of various traits in capturing variation across the entire germplasm. The PCA was performed using WINDOWS STAT statistical software, as recommended by Fujita et al. (1996).

## 3 Results

**Figure 1:**
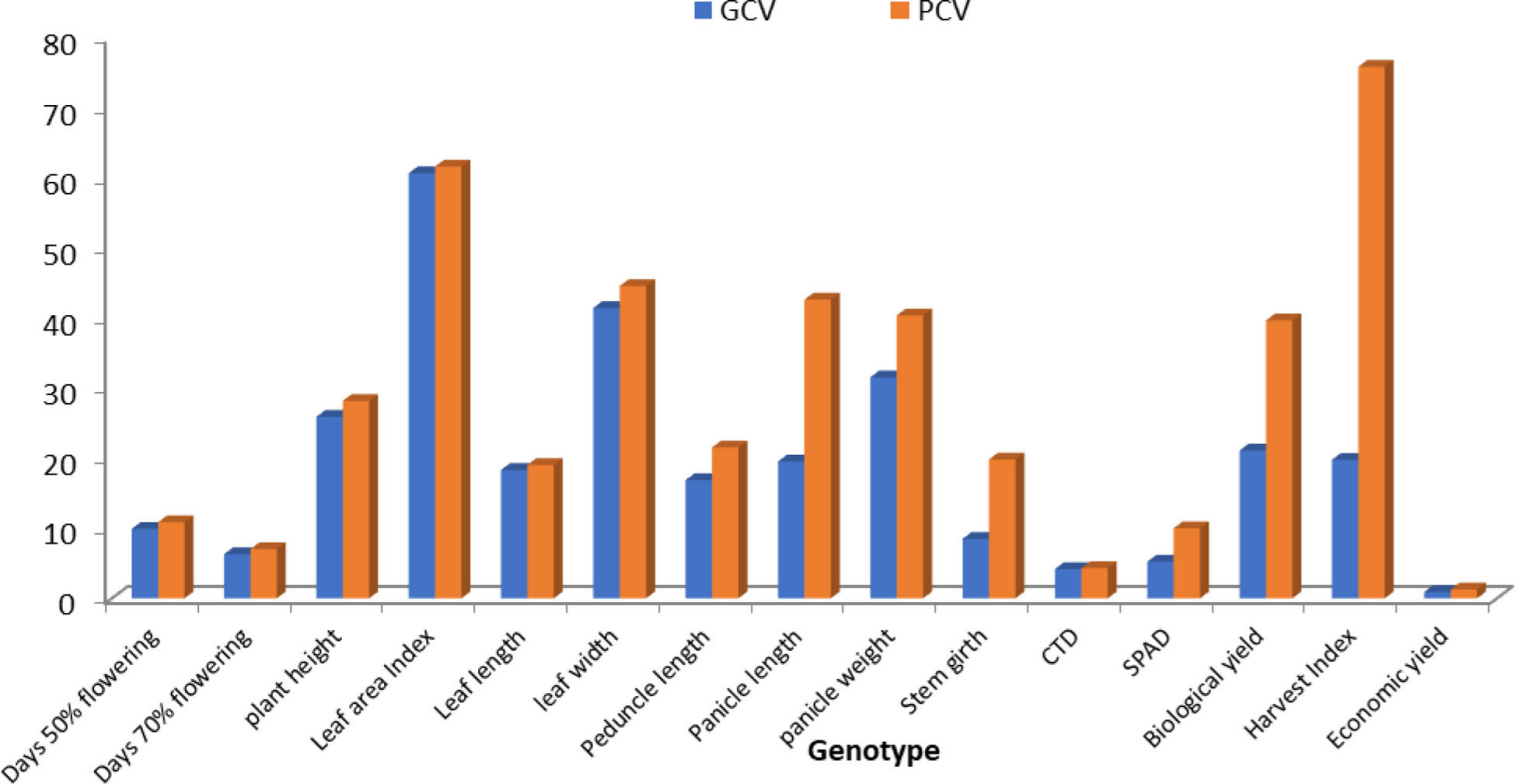
The genotypic and phenotypic coefficient of variation in 50 accessions for different parameters in *S. italica*.

**Figure 2:**
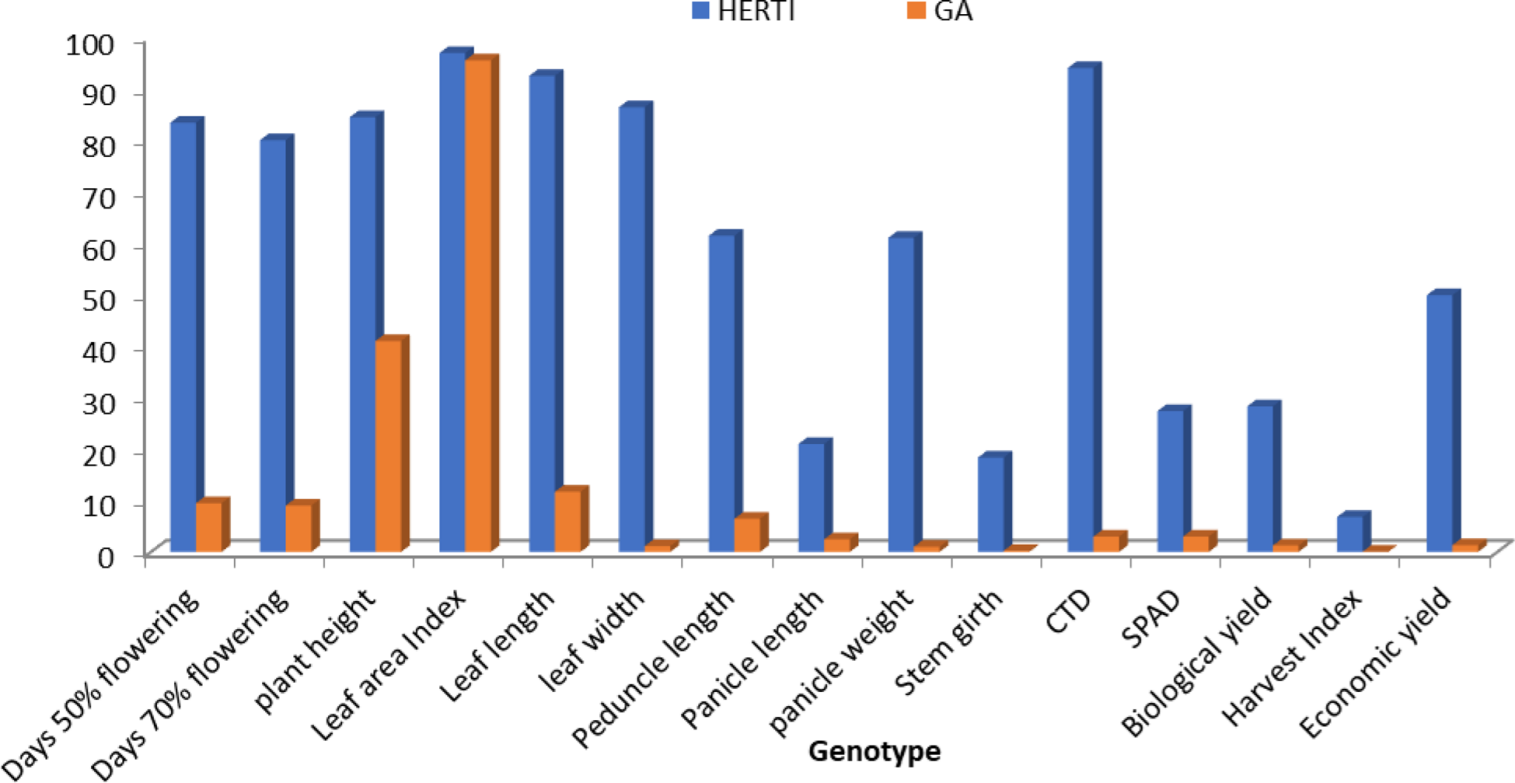
Histogram depicting heritability and genetic advance coefficient of variation for different parameters in *S. italica*.

Accessions showed variability among the quantitative and qualitative characters studied. The genetic parameters of 10 genotypes for 15 characters of foxtail millet were observed. Genotypic variance was high for plant height and low for leaf width. Phenotypic variance was at maximum for plant height and at minimum for leaf width. GCV was at maximum for economic yield and at minimum for conductivity temperature and depth (CTD). PCV was at maximum for economic yield and at minimum for CID. Heritability was at maximum for days of 50% flowering and at minimum for economic yield. Genetic advance (GA) was at maximum for plant height and at minimum for harvest index (**Figures 1**, **2**). Ten accessions were used for calculations of genotypic and phenotypic coefficient of variation for different parameters in *S. Italica* (Singh et al., 2021). GCV and PCV ratios were highest for economic yield, while GCV and PCV ratios were very low for CTD. GCV and PCV ratios for all characters were as follows: days to 50% flowering, 6.50 and 7.03; days to 70% flowering, 5.68 and 5.88; plant height, 20.27 cm and 22.38 cm; leaf width, 10.10 cm and 13.06 cm; leaf length, 10.69 cm and 13.08 cm; pedicle length, 19.07 cm and 20.43 cm; panicle length, 23.97 cm and 25.25 cm; panicle weight, 32.77 g and 37.36 g; leaf area index, 25.23 and 28.86; stem girth, 8.42 cm and 10.31 cm; soil plant analysis development (SPAD), 7.01 and 10.38; CTD, 3.08 and 3.22; harvest index, 31.70% and 40.77%; biological yield, 32.55 g and 38.37 g; and grain yield, 47.24 g and 82.29g.

The heritability and GA ratio was high for the leaf area index and very low for economic yield and the harvest index. The heritability and GA ratios for all characters were as follows: days to 50% flowering, 96.26 and 7.29; days to 70% flowering, 93.31 and 7.29; plant height, 82.00 cm and 42.46 cm; leaf width, 59.86 cm and 0.22 cm; leaf length, 66.80 cm and 6.40 cm; peduncle length, 87.17 cm and 10.09 cm; panicle length, 90.13 cm and 8.10 cm; panicle weight, 76.96 gm and 1.59 gm; leaf area index, 76.43 and 37.96; stem girth, 66.63 cm and 0.39 cm; SPAD, 45.61 nm and 5.26 nm; CTD, 91.43°C and 2.12°C; harvest index, 60.47 gm and 0.21 gm; biological yield, 71.99 gm and 4.26 gm; and economic yield, 32.96 gm and 1.70 gm. Brunda et al. (2014) reported that different crops have contributed to the overall parameters which involve the GCV and PCV traits.

### 3.1 Correlation analysis

The genotypic and phenotypic correlation between yield and yield components and the interrelationships among them are estimated and presented in **Tables 1**, **2**. The qualitative and quantitative characters of the 50 genotypes from 2018 were analyzed to help identify the 10 best genotypes. The same methodology was used to select the five best genotypes from the 10 from 2019.

**Table 1:** Estimation of 50 accessions for the genotypic correlation coefficient 15 yield component in foxtail millet from 2018.

S. No		Days of 50% flowering	Days of 70% flowering	Leaf length (cm)	Leaf width (cm)	Panicle length (cm)	Panicle weight (gm)	Peduncle length (cm)	Plant height (cm)	SPAD	Stem girth (cm)	Biological yield (gm)	CTD	Harvest index (%)	Leaf area index	Economic yield (gm)
1	Days of 50% flowering	1.000	0.474**	0.354**	0.261*	0.302**	0.117	0.413**	0.385**	0.318**	0.016	0.067	0.137	0.080	0.361**	0.085
2	Days of 70% flowering	0.474**	1.000	0.081	0.137	0.232*	0.212*	0.232*	0.277**	0.126	-0.046	-0.091	0.164*	0.170*	0.252*	-0.007
3	Leaf length (cm)	0.354**	0.081	1.000	0.330**	0.343**	0.223*	0.287**	0.447**	0.097	0.224*	0.030	-0.279**	0.125	0.386**	0.140
4	Leaf width (cm)	0.261*	0.137	0.330**	1.000	0.368**	0.250*	0.210*	0.331**	-0.118	0.002	0.311**	-0.012	-0.089	0.875**	0.151
5	Panicle length (cm)	0.302**	0.232*	0.343**	0.368**	1.000	0.026	0.245*	0.494**	-0.101	0.160	0.029	0.074	-0.140	0.295**	-0.032
6	Panicle weight (gm)	0.117	0.212*	0.223*	0.250*	0.026	1.000	0.234*	0.386**	-0.216*	-0.004	0.205*	0.010	0.027	0.298**	0.150
7	Peduncle length (cm)	0.413**	0.232*	0.287**	0.210*	0.245*	0.234*	1.000	0.709**	-0.027	0.645**	0.096	0.171*	-0.140	0.107	-0.022
8	Plant height (cm)	0.385**	0.277**	0.447**	0.331**	0.494**	0.386**	0.709**	1.000	0.052	0.445**	0.234*	0.058	-0.145	0.298**	0.123
9	SPAD	0.318**	0.126	0.097	-0.118	-0.101	-0.216*	-0.027	0.052	1.000	0.032	-0.085	-0.216*	-0.376**	-0.076	-0.369**
10	Stem girth (cm)	0.016	-0.046	0.224*	0.002	0.160	-0.004	0.645**	0.445**	0.032	1.000	0.005	-0.273**	-0.299**	-0.193*	-0.150
11	Biological yield (gm)	0.067	-0.091	0.030	0.311**	0.029	0.205*	0.096	0.234*	-0.085	0.005	1.000	0.252*	-0.312**	0.241*	0.422**
12	CTD	0.137	0.164*	-0.279**	-0.012	0.074	0.010	0.171*	0.058	-0.216*	-0.273**	0.252*	1.000	-0.080	-0.083	-0.021
13	Harvest index (%)	0.080	0.170*	0.125	-0.089	-0.140	0.027	-0.140	-0.145	-0.376**	-0.299**	-0.312**	-0.080	1.000	-0.011	0.557**
14	Leaf area index	0.361**	0.252*	0.386**	0.875**	0.295**	0.298**	0.107	0.298**	-0.076	-0.193*	0.241*	-0.083	-0.011	1.000	0.123
15	Economic yield (gm)	0.085	-0.007	0.140	0.151	-0.032	0.150	-0.022	0.123	-0.369**	-0.150	0.422**	-0.021	0.557**	0.123	1.000

*Significant at the 5% level; ** Significant at 1%.

**Table 2:** Estimation of 10 accessions for the genotypic correlation coefficient 15 yield component in foxtail millet from 2019.

S. No.		Days to 50% flowering	Days to 70% flowering	Peduncle length (cm)	Panicle length (cm)	Panicle weight (gm)	Plant height (cm)	SPAD	Stem girth (cm)	Harvest index (%)	Leaf area index	Leaf length (cm)	Leaf width (cm)	Biological yield (gm)	CTD	Economic yield (cm)
1	Days to 50% flowering	1.000	1.467	-0.225	-0.480*	-0.100	0.030	0.059	-0.656**	0.477*	-0.555*	-0.631**	-0.636**	-0.241	0.692**	0.111
2	Days to 70% flowering	1.467	1.000	-0.475*	-0.703**	-0.072	0.131	-0.077	-0.255	0.680**	-0.637**	-0.695**	-0.424*	-0.329	0.761**	0.145
3	Peduncle length (cm)	-0.225	-0.475*	1.000	0.935**	0.414*	-0.147	-0.226	-0.545*	0.024	0.455*	0.421*	0.378*	0.567*	-0.485*	0.443*
4	Panicle length (cm)	-0.480*	-0.703**	0.935**	1.000	0.503*	0.027	-0.062	-0.399*	-0.029	0.570*	0.624**	0.525*	0.610**	-0.765**	0.436*
5	Panicle weight (gm)	-0.100	-0.072	0.414*	0.503*	1.000	0.635**	0.241	-0.165	0.515*	0.599**	0.652**	0.565*	0.878**	-0.326	0.811**
6	Plant height (cm)	0.030	0.131	-0.147	0.027	0.635**	1.000	0.546*	-0.076	0.615**	0.130	0.391*	0.010	0.609**	-0.363*	0.685**
7	SPAD	0.059	-0.077	-0.226	-0.062	0.241	0.546*	1.000	0.152	0.004	0.062	0.297	-0.135	0.360	0.392*	0.226
8	Stem girth (cm)	-0.656**	-0.255	-0.545*	-0.399*	-0.165	-0.076	0.152	1.000	0.094	-0.180	-0.096	0.007	-0.352	-0.049	-0.192
9	Harvest index (%)	0.477*	0.680**	0.024	-0.029	0.515*	0.615**	0.004	0.094	1.000	-0.227	-0.177	-0.228	0.362*	0.061	0.789**
10	Leaf area index	-0.555*	-0.637**	0.455*	0.570*	0.599**	0.130	0.062	-0.180	-0.227	1.000	1.045	1.082	0.499*	-0.837**	0.097
11	Leaf length (cm)	-0.631**	-0.695**	0.421*	0.624**	0.652**	0.391*	0.297	-0.096	-0.177	1.045	1.000	0.894**	0.590**	-1.048**	0.184
12	Leaf width (cm)	-0.636**	-0.424*	0.378*	0.525*	0.565*	0.010	-0.135	0.007	-0.228	1.082	0.894**	1.000	0.288	-0.959**	-0.044
13	Biological yield (gm)	-0.241	-0.329	0.567*	0.610**	0.878**	0.609**	0.360	-0.352	0.362*	0.499*	0.590**	0.288	1.000	-0.284	0.842**
14	CTD	0.692**	0.761**	-0.485*	-0.765**	-0.326	-0.363*	0.392*	-0.049	0.061	-0.837**	-1.048**	-0.959**	-0.284	1.000	-0.102
15	Economic yield (cm)	0.111	0.145	0.443*	0.436*	0.811**	0.685**	0.226	-0.192	0.789**	0.097	0.184	-0.044	0.842**	-0.102	1.000

**Significant at 1%; *significant at the 5% level.

#### 3.1.1 Genotypic correlation

For the 50 genotypes from 2018, the following genotypic correlations were obtained: days to 70% flowering had a 1% significant genotypic correlation with days to 50% flowering (0.466*); grain yield had a 50% significant genotypic correlation with leaf width (0.184 cm); grain yield had a 1% significant genotypic correlation with biological yield (0.554**); grain yield had a 1% significant genotypic correlation with harvest index (1.059%); and grain yield showed a negative genotypic correlation with SPAD (−0.403), CTD (−0,037), stem girth (−0.326 cm), and panicle length (−0.048 cm).

For the 10 genotypes from 2019, the genotypic correlations are shown in **Table 2** and were as follows: grain yield with plant height (0.915), peduncle length (0.568 cm*), panicle length (0.551 cm), panicle weight (1.028 g), SPAD (0.609), harvest index (1.062%*), and biological yield (1.197 g) showed a 1% significant genotypic correlation; grain yield with leaf width (−0.034 cm), stem girth (−0.275 cm), and CTD (−0.053) showed a negative genotypic correlation; and grain yield with days to 50% flowering (0.109), days to 70% flowering (0.102), plant height (0.015 cm), leaf length (0.249 cm), peduncle length (0.568 cm), panicle length (0.551 cm), leaf area index (1.028), SPAD (0.098), harvest index (0.609%), biological yield (1.062 g), and grain yield (1.197 g) showed a positive genotypic correlation.

#### 3.1.2 Phenotypic correlation

Phenotypic correlations were calculated among the 50 genotypes from 2018. There was 1% significance with days to 70% flowering, plant height, leaf area index, leaf length, leaf width, peduncle length, harvest index, biological yield, grain yield, peduncle weight, and stem girth. Grain yield with days to 70% flowering (−0.015), panicle length (−0.036 cm), stem girth (−0.023 cm), CTD (0.043), and SPAD (0.150) showed a negative phenotypic correlation. Grain yield showed a significantly increased positive correlation with days to 50% flowering (0.071), plant height (0.053 cm), leaf area index (0.099), leaf length (0.117 cm), leaf width (0.084 cm), peduncle length (0.024 cm), panicle weight (0.207 g), biological yield (0.297 g), harvest index (0.506%), and grain yield (1.000 g). Grain yield showed an increased positive phenotypic correlation in 10 genotypes.

The relationship pattern of grain yield with panicle weight, panicle length, leaf width, leaf length, leaf area index, and plant height was comparable at genotypic and phenotypic levels for the 50 genotypes from 2018. A profoundly huge positive affiliation was observed for grain yield per plant with panicle length, leaf width, leaf length, and leaf area index at both the genotypic and phenotypic levels. Comparative outcomes showed that grain yield per plant had a positively huge relationship at the two levels in terms of days to development, panicle length, panicle weight, plant stature, and test weight. Connection examinations likewise give provided data about the relationship between other plant attributes. Plant height had an exceptionally critical positive relationship with number of tillers and panicle length, which was in line with Nirmlakumari and Vetriventhan (2010), and furthermore with panicle width and panicle weight. Leaf area index and panicle weight showed an exceptionally critical positive relationship among themselves. Thus, the determination of both of these qualities increases the chances of improving the other characteristics; therefore, both attributes further improve grain yield.

It is fascinating to note that stem girth shows a positively huge relationship with plant height and peduncle length. Phenotypic correlations in the 10 genotypes collected in 2019 are shown in **Table 2**. Economic yield showed a 5% significant phenotypic correlation with plant height (0.457*), a 1% significant phenotypic correlation with panicle weight (0.615*), harvest index (0.553), and biological yield (0.521*), a negative phenotypic correlation with leaf width (−0.046), stem girth (−0.064), SPAD (−0.113), and CTD (−0.088), and a positive correlation with days to 50% flowering (0.104), days to 70% flowering (0.071), plant height (0.116), leaf length (0.091), peduncle length (0.304), panicle length (0.331), biological yield (0.521), and economic yield (1.000).

### 3.2 Path coefficient analysis

The direct and indirect effects of different yield components in grain yield were calculated through path coefficient analysis at genotypic and phenotypic levels and are shown in **Tables 3**, **4**. The phenotypic and genotypic correlations reveal the extent and direction of association between different characters. These are in agreement with the results obtained by Brunda et al. (2015) in foxtail millet and suggest that selection for these traits indirectly improves grain yield.

**Table 3:** Estimation of 50 accessions for the phenotypic correlation coefficient 15 yield component in foxtail millet from 2018.

S. No.		Days of 50% flowering	Days of 70% flowering	Leaf length (cm)	Leaf width (cm)	Panicle length (cm)	Panicle weight (gm)	Peduncle length (cm)	Plant height (cm)	SPAD	Stem girth (cm)	Biological yield (gm)	CTD	Harvest index (%)	Leaf area index	Economic yield (gm)
1	Days of 50% flowering	1.000	0.454**	0.296**	0.215*	0.268**	0.114	0.347**	0.322**	0.154	0.013	0.063	0.033	0.076	0.309**	0.086
2	Days of 70% flowering	0.454**	1.000	0.053	0.098	0.179*	0.182*	0.152	0.195*	0.037	-0.066	-0.073	0.023	0.151	0.204*	0.002
3	Leaf length (cm)	0.296**	0.053	1.000	0.311**	0.309**	0.201*	0.235*	0.394**	0.054	0.177*	0.028	-0.140	0.115	0.369**	0.128
4	Leaf width (cm)	0.215*	0.098	0.311**	1.000	0.331**	0.225*	0.168*	0.304**	-0.050	-0.007	0.282**	-0.024	-0.082	0.833**	0.131
5	Panicle length (cm)	0.268**	0.179*	0.309**	0.331**	1.000	0.020	0.182*	0.414**	-0.044	0.104	0.060	0.026	-0.141	0.257*	-0.021
6	Panicle weight (gm)	0.114	0.182*	0.201*	0.225*	0.020	1.000	0.184*	0.339**	-0.118	-0.009	0.199*	0.011	0.048	0.263*	0.157
7	Peduncle length (cm)	0.347**	0.152	0.235*	0.168*	0.182*	0.184*	1.000	0.605**	0.049	0.357**	0.068	0.103	-0.125	0.098	-0.028
8	Plant height (cm)	0.322**	0.195*	0.394**	0.304**	0.414**	0.339**	0.605**	1.000	0.043	0.283**	0.198*	0.068	-0.148	0.271**	0.086
9	SPAD	0.154	0.037	0.054	-0.050	-0.044	-0.118	0.049	0.043	1.000	0.023	-0.054	-0.039	-0.210*	-0.053	-0.193*
10	Stem girth (cm)	0.013	-0.066	0.177*	-0.007	0.104	-0.009	0.357**	0.283**	0.023	1.000	0.016	-0.089	-0.198*	-0.130	-0.085
11	Biological yield (gm)	0.063	-0.073	0.028	0.282**	0.060	0.199*	0.068	0.198*	-0.054	0.016	1.000	0.095	-0.310**	0.214*	0.399**
12	CTD	0.033	0.023	-0.140	-0.024	0.026	0.011	0.103	0.068	-0.039	-0.089	0.095	1.000	-0.029	-0.065	-0.015
13	Harvest index (%)	0.076	0.151	0.115	-0.082	-0.141	0.048	-0.125	-0.148	-0.210*	-0.198*	-0.310**	-0.029	1.000	-0.011	0.539**
14	Leaf area index	0.309**	0.204*	0.369**	0.833**	0.257*	0.263*	0.098	0.271**	-0.053	-0.130	0.214*	-0.065	-0.011	1.000	0.116
15	Economic yield (gm)	0.086	0.002	0.128	0.131	-0.021	0.157	-0.028	0.086	-0.193*	-0.085	0.399**	-0.015	0.539**	0.116	1.000

**Significant at 1%; *significant at the 5% level.

**Table 4:** Estimation of 10 accessions for the phenotypic correlation coefficient 15 yield component in foxtail millet from 2019.

	Days to 50% flowering	Days to 70% flowering	Peduncle length (cm)	Panicle length (cm)	Panicle weight (gm)	Plant height (cm)	SPAD	Stem girth (cm)	Harvest index (%)	Leaf area index	Leaf length (cm)	Leaf width (cm)	Biological yield (gm)	CTD	Economic yield (gm)
Days to 50% flowering	1.000	0.556*	-0.217	-0.323	-0.021	0.041	-0.071	-0.251	0.327	-0.314	-0.405*	-0.230	-0.152	0.395*	0.098
Days to 70% flowering	0.556*	1.000	-0.213	-0.390*	-0.067	0.044	-0.120	-0.272	0.389*	-0.280	-0.376*	-0.370*	-0.186	0.276	0.068
Peduncle length (cm)	-0.217	-0.213	1.000	0.879**	0.362*	-0.138	-0.188	-0.356	0.031	0.413*	0.321	0.262	0.538*	-0.217	0.401*
Panicle length (cm)	-0.323	-0.390*	0.879**	1.000	0.485*	0.025	-0.042	-0.277	-0.020	0.540*	0.471*	0.358	0.600**	-0.408*	0.425*
Panicle weight (gm)	-0.021	-0.067	0.362*	0.485*	1.000	0.611**	0.181	-0.087	0.504*	0.578**	0.488*	0.411*	0.857**	-0.229	0.793**
Plant height (cm)	0.041	0.044	-0.138	0.025	0.611**	1.000	0.335	-0.025	0.576**	0.156	0.272	-0.078	0.569*	-0.105	0.650**
SPAD	-0.071	-0.120	-0.188	-0.042	0.181	0.335	1.000	0.135	0.039	0.001	0.042	-0.216	0.234	0.032	0.175
Stem girth (cm)	-0.251	-0.272	-0.356	-0.277	-0.087	-0.025	0.135	1.000	0.060	-0.132	-0.198	0.049	-0.203	0.112	-0.125
Harvest index (%)	0.327	0.389*	0.031	-0.020	0.504*	0.576**	0.039	0.060	1.000	-0.225	-0.182	-0.199	0.351	0.013	0.772**
Leaf area index	-0.314	-0.280	0.413*	0.540*	0.578**	0.156	0.001	-0.132	-0.225	1.000	0.821**	0.746**	0.478*	-0.405*	0.096
Leaf length (cm)	-0.405*	-0.376*	0.321	0.471*	0.488*	0.272	0.042	-0.198	-0.182	0.821**	1.000	0.760**	0.492*	-0.436*	0.136
Leaf width (cm)	-0.230	-0.370*	0.262	0.358	0.411*	-0.078	-0.216	0.049	-0.199	0.746**	0.760**	1.000	0.253	-0.357	-0.037
Biological yield (gm)	-0.152	-0.186	0.538*	0.600**	0.857**	0.569*	0.234	-0.203	0.351	0.478*	0.492*	0.253	1.000	-0.101	0.831**
CTD	0.395*	0.276	-0.217	-0.408*	-0.229	-0.105	0.032	0.112	0.013	-0.405*	-0.436*	-0.357	-0.101	1.000	-0.032
Economic yield (gm)	0.098	0.068	0.401*	0.425*	0.793**	0.650**	0.175	-0.125	0.772**	0.096	0.136	-0.037	0.831**	-0.032	1.000

**Significant at 1%; *significant at the 5% level.

#### 3.2.1 Genotypic path correlation

Genotypic path correlation revealed a highly positive direct effect of panicle length. Days to 50% flowering, plant height, leaf width, biological yield, economic yield, and harvest index showed a negative genotypic path coefficient analysis with plant height (−0.1375), leaf width (−0.1275), peduncle length (−0.0190), panicle length (−0.1401), panicle weight (−0.051), stem girth (−0.6868), CTD (−0.1372), and SPAD (0.3700). Harvest index showed a significant increase and positive correlation coefficient analysis with days to 50% flowering (0.0979), days to 70% flowering (0.2048), leaf area index (0.0218), leaf length (0.2456), biological yield (0.0109), and economic yield (0.7547).

#### 3.2.2 Phenotypic path correlation

The phenotypic path in the 50 genotypes collected in 2018 is shown in **Table 5** and revealed a highly positive direct effect of plant height, leaf length, leaf width, panicle length, and biological yield. Harvest index showed a negative phenotypic path correlation with plant height (−0.0630), leaf area index (−0.0032), leaf width (−0.0431), peduncle length (−0.0561), panicle length (−0.0988), stem girth (−0.0201), CTD (−0.281), SPAD (−0.1502), and biological yield (−0.2820), and a positive phenotypic path coefficient analysis with days to 50% flowering (0.0419), days to 70% flowering (0.0582), leaf length (0,0740), and panicle weight (0.0359).

**Table 5:** Genotypic path of 15 yield component traits in 50 foxtail millet accessions from 2018.

	Days of 50% flowering	Days of 70% flowering	Leaf length (cm)	Leaf width (cm)	Panicle length (cm)	Panicle weight (gm)	Peduncle length (cm)	Plant height (cm)	SPAD	Stem girth (cm)	Biological yield (cm)	CTD	Harvest Index (%)	Leaf area index	Economic yield (gm)
Days of 50% flowering	0.123	0.059	0.044	0.032	0.037	0.015	0.051	0.048	0.039	0.002	0.008	0.017	0.010	0.045	0.085
Days of 70% flowering	-0.012	-0.025	-0.002	-0.003	-0.006	-0.005	-0.006	-0.007	-0.003	0.001	0.002	-0.004	-0.004	-0.006	-0.007
Leaf length (cm)	-0.008	-0.002	-0.023	-0.008	-0.008	-0.005	-0.007	-0.011	-0.002	-0.005	-0.001	0.007	-0.003	-0.009	0.140
Leaf width (cm)	0.073	0.038	0.092	0.279	0.103	0.070	0.058	0.092	-0.033	0.000	0.087	-0.003	-0.025	0.244	0.151
Panicle length (cm)	-0.014	-0.011	-0.016	-0.017	-0.047	-0.001	-0.012	-0.023	0.005	-0.008	-0.001	-0.004	0.007	-0.014	-0.032
Panicle weight (gm)	-0.010	-0.017	-0.018	-0.020	-0.002	-0.081	-0.019	-0.031	0.018	0.000	-0.017	-0.001	-0.002	-0.024	0.150
Peduncle length (cm)	-0.010	-0.005	-0.007	-0.005	-0.006	-0.005	-0.023	-0.017	0.001	-0.015	-0.002	-0.004	0.003	-0.003	-0.022
Plant height (cm)	0.096	0.069	0.112	0.083	0.124	0.097	0.178	0.250	0.013	0.111	0.059	0.015	-0.036	0.075	0.123
SPAD	-0.061	-0.024	-0.019	0.023	0.019	0.042	0.005	-0.010	-0.192	-0.006	0.016	0.042	0.072	0.015	-0.369**
Stem girth (cm)	-0.003	0.009	-0.044	0.000	-0.031	0.001	-0.126	-0.087	-0.006	-0.196	-0.001	0.053	0.059	0.038	-0.150
Biological yield (cm)	0.043	-0.059	0.019	0.200	0.019	0.132	0.062	0.151	-0.055	0.003	0.644	0.162	-0.201	0.155	0.422**
CTD	-0.039	-0.046	0.079	0.003	-0.021	-0.003	-0.048	-0.016	0.061	0.077	-0.071	-0.282	0.023	0.024	-0.021
Harvest index (gm)	0.052	0.111	0.081	-0.058	-0.091	0.018	-0.091	-0.095	-0.245	-0.195	-0.203	-0.052	0.651	-0.007	0.557**
Leaf area index	-0.147	-0.103	-0.158	-0.357	-0.121	-0.122	-0.044	-0.122	0.031	0.079	-0.098	0.034	0.005	-0.408	0.123
Economic yield (gm)	0.085	-0.007	0.140	0.151	-0.032	0.150	-0.022	0.123	-0.369**	-0.150	0.422**	-0.021	0.557**	0.123	1.000
Partial R^2^	0.010	0.000	-0.003	0.042	0.002	-0.012	0.001	0.031	0.071	0.029	0.272	0.006	0.363	-0.050	

**Significant at 1%.

For the 10 genotypes collected in 2019, genotypic path coefficient analysis is shown in **Table 6**, which reveals the highly positive direct effect of harvest index, panicle weight, and plant height. Genotypic path coefficient analysis showed a positive genotypic path for days to 50% flowering (2.364), leaf length (0.427), panicle length (0.741), and leaf area index (2.747). Biological yield showed a positive genotypic path coefficient analysis with days to 50% flowering (0.234), days to 70% flowering (0.296), stem girth (0.366), and CTD (0.236). The immediate and roundabout impacts of various yield segments on grain yield were determined through weight examination at phenotypic and genotypic levels. This examination uncovered the high and immediate impact of plant height, peduncle length, and leaf width on grain yield per plant in 50 germplasm assortments. This demonstrates a genuine connection between these characters with grain yield per plant and the direct determination of these attributes helps to improve grain yield per plant. Comparable investigations of grain yield were carried out at the genotypic and phenotypic level in terms of panicle weight, test weight, and straw weight. Weight examination showed that plant height and peduncle length significantly and immediately affected grain yield. This positive direct impact of plant stature and peduncle length on grain yield suggests that the biomass of a plant should be built up to increase yield. The weight investigation revealed that the immediate impact of plant height, leaf length, leaf width, and panicle length on grain yield was positive. For this characteristic to produce the desired results, it would seem that determination must be focused in a particular direction.

**Table 6:** Genotypic path of 15 yield component traits in 10 foxtail millet accessions from 2019.

	Days of 50% flowering	Days of 70% flowering	Leaf length (cm)	Leaf width (cm)	Panicle length (cm)	Panicle weight (gm)	Peduncle length (cm)	Plant height (cm)	SPAD	Stem girth (cm)	Biological yield (cm)	CTD	Harvest index (%)	Leaf area index	Economic yield (gm)
Days of 50% flowering	0.007	0.010	-0.002	-0.003	-0.001	0.000	0.000	-0.005	0.003	-0.004	-0.004	-0.004	-0.002	0.005	0.111
Days of 70% flowering	-0.040	-0.027	0.013	0.019	0.002	-0.004	0.002	0.007	-0.018	0.017	0.019	0.012	0.009	-0.021	0.145
Leaf length (cm)	0.014	0.030	-0.063	-0.058	-0.026	0.009	0.014	0.034	-0.002	-0.029	-0.026	-0.024	-0.035	0.030	0.443
Leaf width (cm)	-0.114	-0.167	0.222	0.237	0.119	0.007	-0.015	-0.095	-0.007	0.135	0.148	0.124	0.145	-0.181	0.436
Panicle length (cm)	-0.017	-0.012	0.070	0.085	0.170	0.108	0.041	-0.028	0.087	0.102	0.111	0.096	0.149	-0.055	0.811
Panicle weight (gm)	-0.001	-0.005	0.005	-0.001	-0.023	-0.036	-0.020	0.003	-0.022	-0.005	-0.014	0.000	-0.022	0.013	0.685
Peduncle length (cm)	-0.001	0.001	0.003	0.001	-0.003	-0.007	-0.013	-0.002	0.000	-0.001	-0.004	0.002	-0.005	-0.005	0.226
Plant height (cm)	-0.003	-0.001	-0.002	-0.002	-0.001	0.000	0.001	0.004	0.000	-0.001	0.000	0.000	-0.002	0.000	-0.192
SPAD	0.235	0.336	0.012	-0.014	0.254	0.304	0.002	0.047	0.494	-0.112	-0.087	-0.113	0.179	0.030	0.789
Stem girth (cm)	0.111	0.127	-0.091	-0.114	-0.120	-0.026	-0.012	0.036	0.045	-0.200	-0.209	-0.216	-0.100	0.167	0.097
Biological yield (cm)	-0.024	-0.027	0.016	0.024	0.025	0.015	0.011	-0.004	-0.007	0.040	0.039	0.034	0.023	-0.040	0.184
CTD	0.054	0.036	-0.032	-0.045	-0.048	-0.001	0.011	-0.001	0.019	-0.092	-0.076	-0.085	-0.025	0.081	-0.044
Harvest index (gm)	-0.129	-0.176	0.303	0.326	0.469	0.325	0.192	-0.188	0.193	0.267	0.315	0.154	0.534	-0.152	0.842
Leaf area index	0.018	0.019	-0.012	-0.019	-0.008	-0.009	0.010	-0.001	0.002	-0.021	-0.026	-0.024	-0.007	0.025	-0.102
Economic yield (gm)	0.111	0.145	0.443	0.436	0.811	0.685	0.226	-0.192	0.789	0.097	0.184	-0.044	0.842	-0.102	1.000
Partial R^2^	0.001	-0.004	-0.028	0.103	0.138	-0.025	-0.003	-0.001	0.390	-0.020	0.007	0.004	0.450	-0.003	

The immediate impact of days to 70% flowering on grain yield was low and negative in both genotype and phenotype. The immediate impact of stem girth on grain yield was positive and low, which corroborates the findings of Nirmlakumari and Vetriventhan (2010). This positive direct impact of plant height on grain yield is attractive as it offers a way to increase straw and grain yield. The immediate impact of test weight on grain yield per plant was positive and high in both seasons, which demonstrates the genuine relationship between these attributes and a straightforward method for increasing grain yield. There is a tendency to believe that the determination of panicle length in foxtail millet will lead to plant height, stem girth, and leaf length being targeted to expand grain yield per plant. In light of the consequences of the weight examination, there is a tendency to infer that increasing characters such as panicle length, plant height, and stem girth, which had a positive connection with and direct impact on yield, will improve yield. Henceforth, lavish plants with enormous panicles, increased grain weight, and high panicle weight might bring about a better return in genotypes of foxtail millet.

The phenotypic path in the 10 genotypes collected in 2019 is shown in **Table 7** and revealed a highly positive direct effect of plant height, harvest index, and biological yield. Biological yield showed a positive phenotypic path with plant height (0.037), leaf width (0.012), leaf length (0.031), pedicle length (-0.034), panicle length (0.036), panicle weight (0.052), and SPAD (0.027) and a negative phenotypic path with days to 70% flowering (−0.013), days to 50% flowering (−0.016), stem girth (−0.016), CTD (−0.011), harvest index (0.013), and biological yield. (0.016). Path analysis indicated that plant height and harvest index had a highly positive direct effect on grain yield (**Table 8**). This positive direct effect of plant height and biological yield on economic yield provides scope to increase the biomass of plants with increased yield.

**Table 7:** Phenotypic path of 15 yield component traits in 50 foxtail millet accessions from 2018.

	Days of 50% flowering	Days of 70% flowering	Leaf length (cm)	Leaf width (cm)	Panicle length (cm)	Panicle weight (gm)	Peduncle length (cm)	Plant height (cm)	SPAD	Stem girth (cm)	Biological yield (cm)	CTD	Harvest index (%)	Leaf area index	Economic yield (gm)
Days of 50% flowering	0.025	0.014	-0.005	-0.008	-0.001	0.001	-0.002	-0.006	0.008	-0.008	-0.010	-0.006	-0.004	0.010	0.098
Days of 70% flowering	-0.022	-0.040	0.008	0.016	0.003	-0.002	0.005	0.011	-0.015	0.011	0.015	0.015	0.007	-0.011	0.068
Leaf length (cm)	0.017	0.017	-0.078	-0.069	-0.028	0.011	0.015	0.028	-0.002	-0.032	-0.025	-0.020	-0.042	0.017	0.401
Leaf width (cm)	-0.064	-0.078	0.175	0.199	0.096	0.005	-0.008	-0.055	-0.004	0.107	0.094	0.071	0.119	-0.081	0.425
Panicle length (cm)	-0.003	-0.008	0.044	0.058	0.120	0.073	0.022	-0.010	0.061	0.070	0.059	0.049	0.103	-0.028	0.793
Panicle weight (gm)	-0.002	-0.002	0.005	-0.001	-0.023	-0.037	-0.012	0.001	-0.021	-0.006	-0.010	0.003	-0.021	0.004	0.650
Peduncle length (cm)	0.002	0.003	0.004	0.001	-0.004	-0.007	-0.021	-0.003	-0.001	0.000	-0.001	0.005	-0.005	-0.001	0.175
Plant height (cm)	0.004	0.004	0.005	0.004	0.001	0.000	-0.002	-0.014	-0.001	0.002	0.003	-0.001	0.003	-0.002	-0.125
SPAD	0.161	0.191	0.015	-0.010	0.248	0.283	0.019	0.030	0.492	-0.111	-0.090	-0.098	0.173	0.006	0.772
Stem girth (cm)	0.050	0.045	-0.066	-0.086	-0.092	-0.025	0.000	0.021	0.036	-0.159	-0.131	-0.119	-0.076	0.065	0.096
Biological yield (cm)	-0.012	-0.011	0.009	0.014	0.014	0.008	0.001	-0.006	-0.005	0.024	0.029	0.022	0.014	-0.013	0.136
CTD	0.023	0.036	-0.026	-0.035	-0.040	0.008	0.021	-0.005	0.019	-0.073	-0.074	-0.098	-0.025	0.035	-0.037
Harvest index (gm)	-0.089	-0.109	0.316	0.352	0.503	0.334	0.137	-0.119	0.206	0.281	0.289	0.149	0.587	-0.059	0.831
Leaf area index	0.010	0.007	-0.006	-0.010	-0.006	-0.003	0.001	0.003	0.000	-0.010	-0.011	-0.009	-0.003	0.025	-0.032
Economic yield (gm)	0.098	0.068	0.401	0.425	0.793	0.650	0.175	-0.125	0.772	0.096	0.136	-0.037	0.831	-0.032	1.000
Partial R^2^	0.002	-0.003	-0.031	0.084	0.095	-0.024	-0.004	0.002	0.380	-0.015	0.004	0.004	0.488	-0.001	

**Table 8:** Phenotypic path of 15 yield component traits in 10 foxtail millet accessions from 2019.

	Days of 50% flowering	Days of 70% flowering	Leaf length (cm)	Leaf width (cm)	Panicle length (cm)	Panicle weight (gm)	Peduncle length (cm)	Plant height (cm)	SPAD	Stem girth (cm)	Biological yield (cm)	CTD	Harvest index (%)	Leaf area index	Economic yield (gm)
Days of 50% flowering	0.013	0.006	0.004	0.003	0.003	0.001	0.004	0.004	0.002	0.000	0.001	0.000	0.001	0.004	0.086
Days of 70% flowering	-0.038	-0.083	-0.004	-0.008	-0.015	-0.015	-0.013	-0.016	-0.003	0.006	0.006	-0.002	-0.013	-0.017	0.002
Leaf length (cm)	-0.007	-0.001	-0.024	-0.007	-0.007	-0.005	-0.006	-0.009	-0.001	-0.004	-0.001	0.003	-0.003	-0.009	0.128
Leaf width (cm)	0.008	0.004	0.012	0.038	0.013	0.009	0.006	0.012	-0.002	0.000	0.011	-0.001	-0.003	0.032	0.131
Panicle length (cm)	0.008	0.005	0.009	0.010	0.029	0.001	0.005	0.012	-0.001	0.003	0.002	0.001	-0.004	0.008	-0.021
Panicle weight(gm)	-0.001	-0.002	-0.002	-0.002	0.000	-0.011	-0.002	-0.004	0.001	0.000	-0.002	0.000	-0.001	-0.003	0.157
Peduncle length (cm)	-0.012	-0.005	-0.008	-0.006	-0.006	-0.006	-0.035	-0.021	-0.002	-0.012	-0.002	-0.004	0.004	-0.003	-0.028
Plant height (cm)	0.036	0.022	0.044	0.034	0.046	0.038	0.068	0.112	0.005	0.032	0.022	0.008	-0.017	0.030	0.086
SPAD	0.000	0.000	0.000	0.000	0.000	0.000	0.000	0.000	-0.003	0.000	0.000	0.000	0.001	0.000	-0.193*
Stem girth (cm)	0.000	-0.002	0.004	0.000	0.002	0.000	0.008	0.006	0.001	0.022	0.000	-0.002	-0.004	-0.003	-0.085
Biological yield (cm)	0.039	-0.045	0.017	0.173	0.037	0.122	0.042	0.122	-0.034	0.010	0.616	0.059	-0.191	0.132	0.399**
CTD	-0.002	-0.001	0.008	0.001	-0.002	-0.001	-0.006	-0.004	0.002	0.005	-0.006	-0.058	0.002	0.004	-0.015
Harvest index (gm)	0.059	0.116	0.088	-0.063	-0.108	0.037	-0.096	-0.113	-0.161	-0.152	-0.237	-0.022	0.765	-0.008	0.539**
Leaf area index	-0.015	-0.010	-0.018	-0.041	-0.013	-0.013	-0.005	-0.013	0.003	0.006	-0.011	0.003	0.001	-0.050	0.116
Economic yield (gm)	0.086	0.002	0.128	0.131	-0.021	0.157	-0.028	0.086	-0.193*	-0.085	0.399**	-0.015	0.539**	0.116	1.000
Partial R^2^	0.001	0.000	-0.003	0.005	-0.001	-0.002	0.001	0.010	0.001	-0.002	0.246	0.001	0.412	-0.006	

**Significant at 1%; *significant at the 5% level.

### 3.3 Principal component analysis

**Figure 3:**
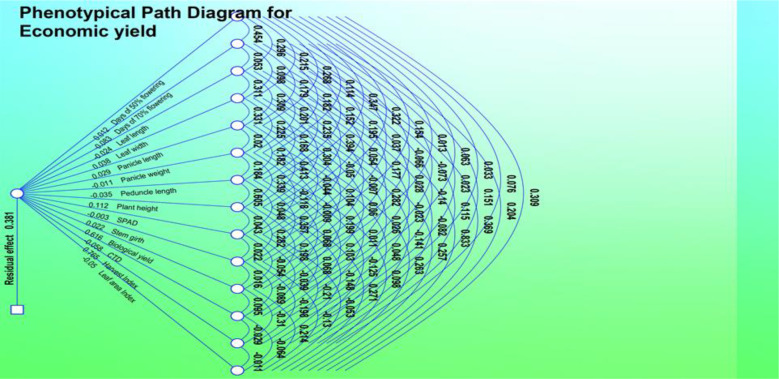
Phenotypic path diagram for grain yield of 15 yield component in 50 foxtail millet accessions collected in 2018.

PCA reduces a very large series of data into a smaller number of components by looking for groups with very strong intercorrelations with a set of variables, and each component is explained as a percentage of variation to the overall variability. The first main component explains most of the overall population variation, followed by subsequent components for huge data, PCA was used to reduce the multivariate data to determine the importance and contribution of each component to the total variance. From the data shown in **Figure 3**, total variation could be 100% explained by 15 principal components (PCs). The PC eigenvalues of 50 genotypes were calculated and represented F1 to F15. F1 was the largest contributing PC, followed by F2, F3, F4, F5, F6, F7, F8, F9, F10, F11, F12, F13, F14, and F15. PC F1 was the most important contributing character, with an eigen value of 3.590, variability of 23.931%, and a cumulative variance of 23.931%. For the entire germplasm, PC F1 mainly separated accessions with the following 14 traits: days of 50% flowering (0.324), days of 70% flowering (0.231), leaf length (0.318), leaf width (0.362), panicle length (0.291), panicle weight (0.242), peduncle length (0.327), plant height (0.416), SPAD (0.004), stem girth (0.128), biological yield (0.167), CTD (0.039), economic yield (0.103), and leaf area index (0.360), which had the highest loadings in PC F1, indicating the significant importance of this component. These traits explained the largest portion of the variability.

**Figure 4:**
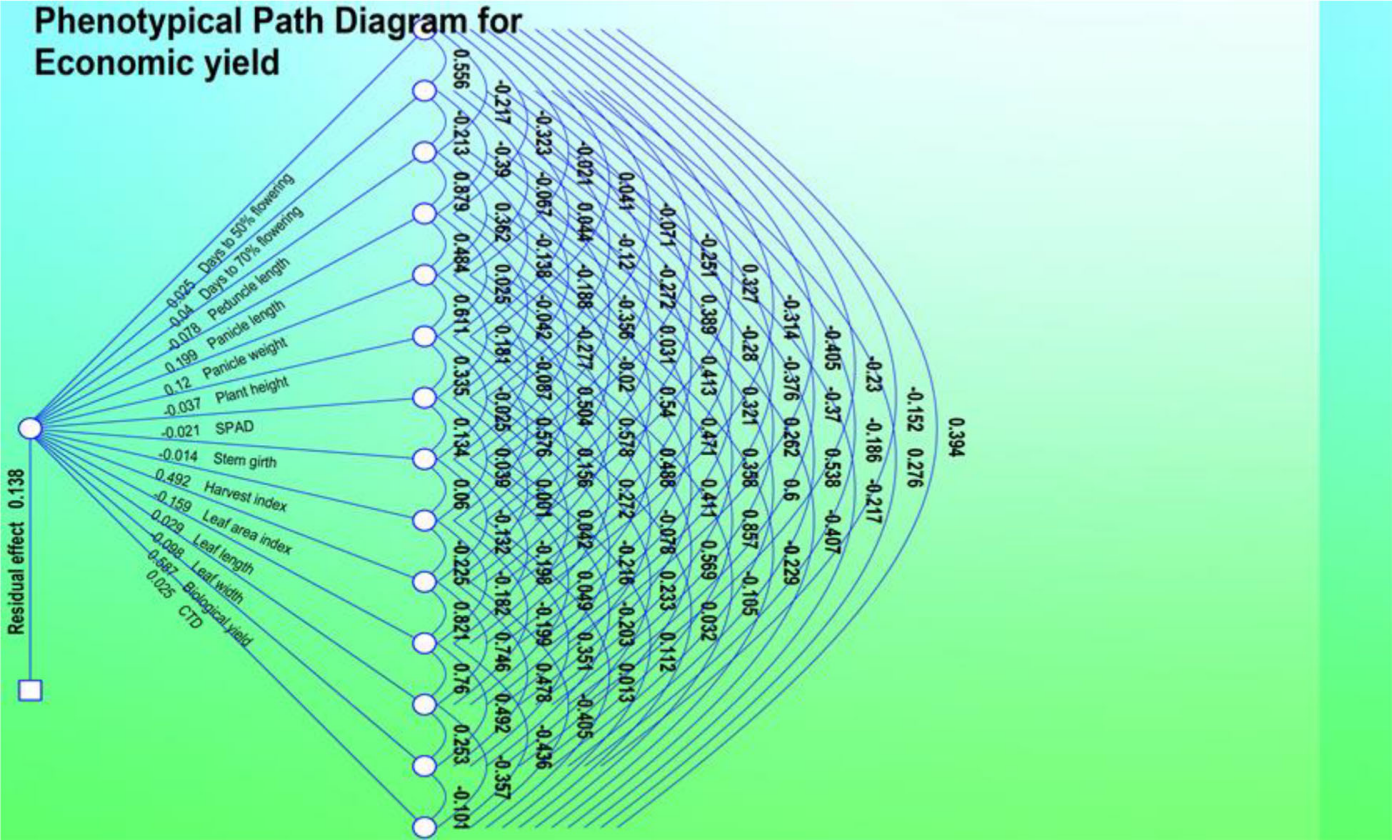
Phenotypic path diagram for grain yield of 15 yield component in 10 foxtail millet accessions collected in 2019.

**Figure 5:**
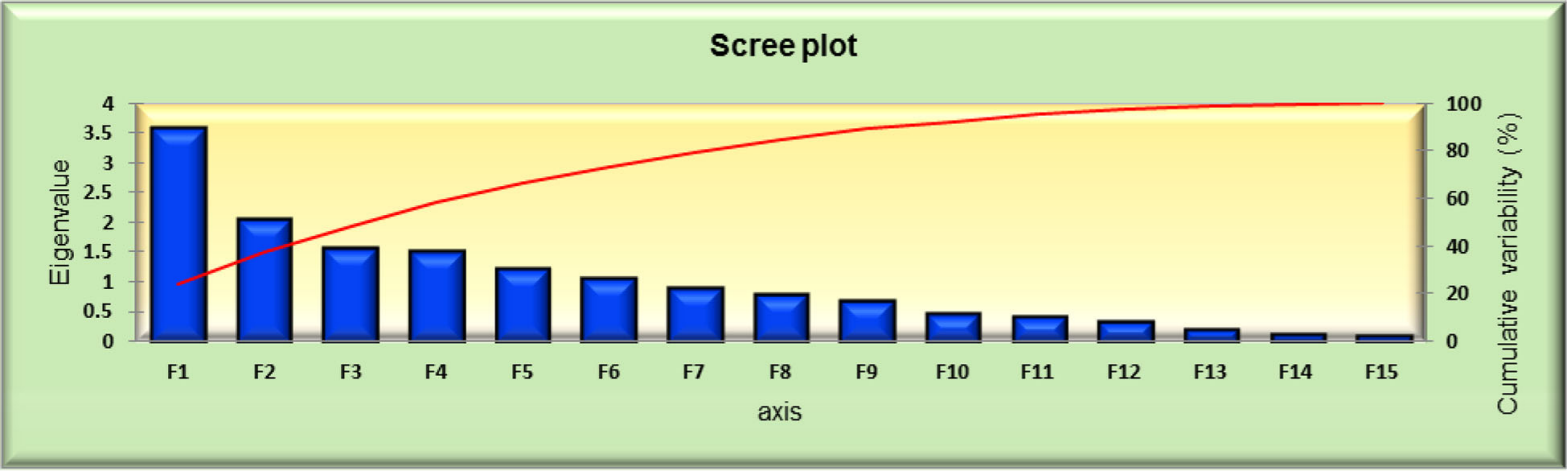
PCA scree plot series 1 and 2 for 50 foxtail millet accessions collected in 2018.

**Figure 6:**
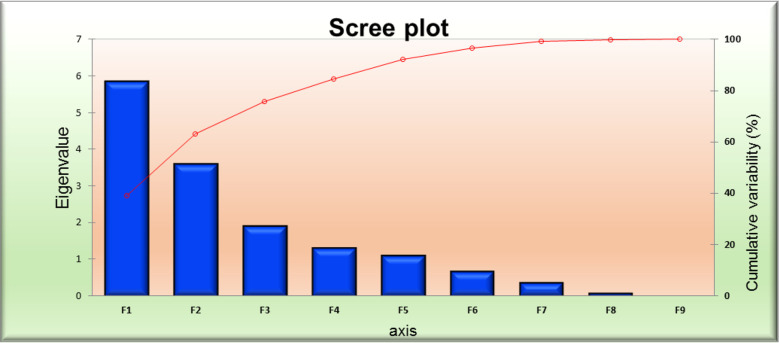
PCA scree plot series 1 and 2 for 10 foxtail millet accessions collected in 2019.

The results of the PCA are shown in **Figures 3**–**6**. PC F2 had an eigenvalue of 2.069, a variability of 13.794%, and a cumulative variability of 37.725%. PC F2 mainly separates accessions with eight traits: leaf length (0.005), leaf width (0.194), panicle weight (0.155), biological yield (0.190), CTD (0.044), economic yield (0.499), harvest index (0.416), and leaf area index (0.253), indicating their significant importance for these components. The remaining characters contributed negatively to the first component. The main traits for PC F3 were days of 50% flowering (0.230), days of 70% flowering (0.261), leaf length (0.225), panicle length (0.018), peduncle length (0.064), plant height (0.017), SPAD (0.073), stem girth (0.108), economic yield (0.165), and harvest Index (0.557). PC F4 had an eigenvalue of 1.512, a variability of 10.080%, and a cumulative variability (CV) of 58.221%. PC F5 had an eigenvalue of 1.235, a variability of 8.236%, and a CV of 66.457%. PC F6 had an eigenvalue of 1.050, a variability of 7.002%, and a CV of 73.458%. PC F7 had an eigenvalue of 0.902, a variability of 6.014%, and a CV of 79.473%. PC F8 had an eigenvalue of 0.798, a variability of 5.323%, and a CV of 84.796%. PC F9 had an eigenvalue of 0.671, a variability of 4.471%, and a CV of 89.267%. PC F10 had an eigenvalue of 0.460, a variability of 3.065%, and a CV of 92.332%. PC F11 had an eigenvalue of 0.419, a variability of 2.795%, and a CV of 95.128%. PC F12 had an eigenvalue of 0.323, a variability of 2.156%, and a CV of 97.284%. PC F13 had an eigen value of 0.204, a variability of 1.360%, and a CV of 98.643%. PC F14 had an eigenvalue of 0.109, a variability of 0.729%, and a CV of 99.373%. For the PCA for the entire germplasm, six traits (days to 50% flowering, plant height, peduncle length, panicle weight, leaf length, and economic yield) explained most of the variance in the first five principal components, indicating their importance for the characterization of foxtail millet germplasm accessions.

**Figure 7:**
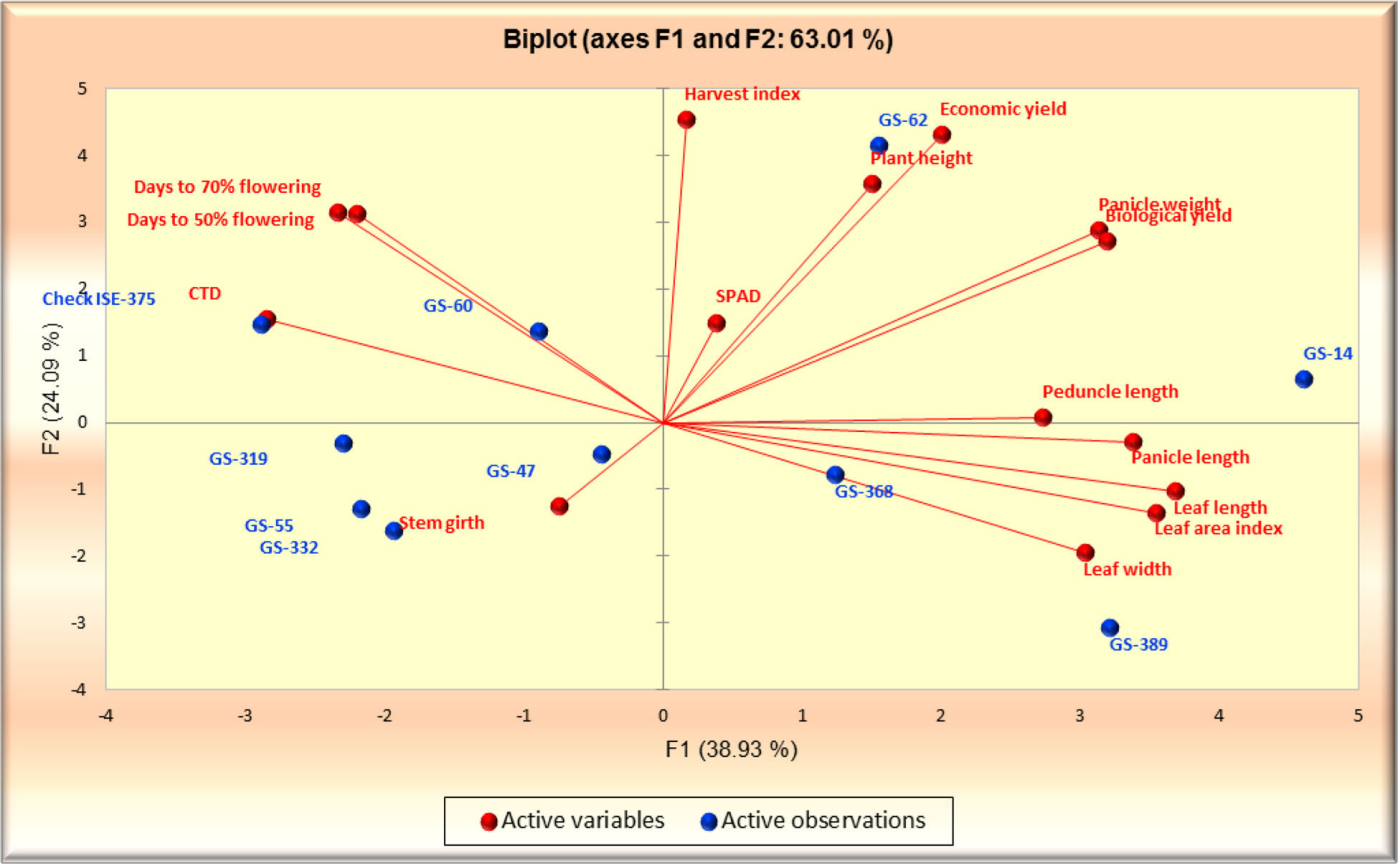
PCA biplot series 1 and 2 for 10 foxtail millet accessions collected in 2019.

The principal component analysis featured the eigenvalue, variability (%), and cumulative variability (%) with respect to principal components F1 – F9. Component F1 was the largest contributing principal component followed by F2, F3, F4, F5, F6, F7, F8, and F9. PC F1 had the highest eigenvalue (5.839), with a variability of 38.926% and cumulative variability of 38.926%. PC F2 had the second highest eigenvalue (3.613), with a variability of 24.087%, and a cumulative variability of 63.013%. PC F3 had the third highest values, followed by F4 and F5. PCs F9 and F8 had the lowest eigenvalues. PC F9 had an eigenvalue of 0.031, a variability of 0.204%, and a cumulative variability of 100.00%. PC F8 had an eigenvalue of 0.091, a variability of 0.607%, and a cumulative variability of 99.796%. PC F6 and F7 had moderate ECV values (**Table 4**). In the scree plot, the red line represents cumulative variability (%) with respect to PCs F1 to F9. In the biplot graph, the PCA in general confirmed the groupings, which were obtained through cluster analysis. The results of PCA are shown in **Figure 7**. The first two PCs with an eigenvalue of >1 accounted for 63.013% of the total variance. Accessions GS-14 and GS-62 had more PCA value than the other genotype principal components.

The breeding of high-yielding varieties is dependent on the yield-contributing morphological features, and we chose a small number of key traits with a favorable association. F lag leaf area, plant height, peduncle length, and tiller count per plant are major morphological yield contributing factors that are positively connected with yield per plant (Eberhart and Russel, 1966). This experiment suggested that high yielding foxtail millet accessions can be selected through indirect selection of panicle length, panicle weight, stem girth, and economic yield. The accessions GS-14 and GS-62 demonstrated the best performance for the majority of yield-related parameters, and hence can be relevant for further investigation in other regions of Uttar Pradesh similar to the Naini regions.

## 4 Discussion

The short-term strategy for identifying foxtail millet genotypes rich in grain nutrients to fulfil the urgent requirement of target micronutrient and protein-deficient populations is to analyze, detect, and explore existing genetic diversity. Significant heterogeneity in all grain nutrients was identified in the foxtail millet core collection, implying that there is plenty of room for selecting nutrient-rich accessions for use in breeding approaches. Field trials were carried out at a variety of sites and across two seasons (2018 and 2019). Estimates of variability, heritability, genetic advance, genotypic correlation coefficient, phenotypic correlation coefficient, genotypic route, and phenotypic path were obtained from the data. Significant differences were observed in Kharif Season-2018 to Kharif Season-2019 among the genotypes for all the characters studied. The results showed that analysis of variance revealed significant differences for most of the traits, including days to 50% flowering, days to 75% maturity, plant height, leaf length, leaf width, leaf area index, panicle length, panicle weight, biological yield, economic yield, harvest index, and test weight, indicating that all genotypes were genetically diverse for most of the traits. GCV estimations for grain yield were the highest, followed by panicle length and biological yield. Leaf length had the highest PCV estimates, followed by plant height and leaf width. Leaf length had the highest heritability, followed by pedicle length. Biological yield, economic yield, and agricultural yield all showed significant genetic progress. H arvest index, leaf breadth, panicle weight, panicle length, and leaf area index were all moderately recorded. L ow GCV and PCV were recorded in leaf length and days to 50% flowering. In conclusion, the genotypes Kangni-1, Kangni-7, Kangni-6, Kangni-5, and Kangni-4 showed the best mean performance in the agroclimatic conditions of Allahabad. The direct influence of biological yield on economic yield per plant was positive and high in both years, which indicates that this feature has a true link and that direct selection using this attribute will be effective.

In foxtail millet, the direct selection of biological yield resulted in the simultaneous indirect selection of several panicles, panicle length, pedicle length, panicle weight, number of productive tillers, and biological yield for higher economic production per plant. Seed yield per plant was found to be positively and significantly linked with biological yield, panicle weight, harvest index, leaf length, leaf area index, leaf breadth, plant height, days to flowering, and days to maturity. This suggests that these traits are mostly driven by additive gene action, and thus direct selection for these traits will result in increased grain yield. Similar results were reported by Vinsonias (2018) for plant height and panicle length for plant height for 1,000 g grain weight and flag leaf blade length (Brunda et al., 2015).

Characters such as leaf length, days to 50% flowering, and days to 75% maturity demonstrated high heritability combined with moderate genetic advance, indicating that there is a greater chance of inheritance from progeny to offspring, and thus these characters should be prioritized for effective selection. Earlier studies have also reported a significantly positive association of biological yield per plant with productive panicle and peduncle length (Brunda et al., 2014; Kumar et al., 2015; Kavya et al., 2017). The positive correlation of yield with other characters indicated that all these characters could be simultaneously improved and that an increase in any one of them would lead to an improvement of other characters. Selection criteria should consider all these characters for to improve biological yield in foxtail millet. The PCA data reduction technique extracts the most important information from the data table (Islam, 2004), compresses the size of the data set by keeping only the important information (Maji and Shaibu, 2012), simplifies the description of the data set (Adams, 1995), and analyzes the structure of the observations and the variables (Amy and Pritts, 1991). Often, only the important information needs to be extracted from a data matrix, and the number of components that are needed should be considered. This problem can be overcome by using some guidelines. The first procedure is to plot the eigenvalues according to their size and to see whether there is a point in the graph (elbow) such that the slope of the graph goes from steep to flat and keep only the components that occur before the elbow. This procedure is called the scree or elbow test (Cattell, 1966; Jolliffe, 2002). Germplasm evaluation and characterization for plant breeders and multivariate statistical analysis estimate the genotypic and phenotypic parameters. The characteristics described in the list of pre-harvest and post-harvest observations were used for selecting the five best genotypes. PCV values were higher than GCV values, which indicates the effect of the environment on the expression of characters. These results are based on data for 2 years. The genotypes Kangni-1 (GS-14), Kangni-7 (GPF-7), Kangni-6 (GS-55), Kangni-5 (GS-389), and Kangni-4 (GS-368) cannot be found anywhere except SHUATS. That is why these genotypes are named by SHUATS. These five best genotypes will be further analyzed through biochemical trait analysis (Singh et al., 2022).

## 5 Conclusion

The present study found substantial diversity in the 50 genotypes of foxtail millet investigated for several agro-morphological variables that might be exploited efficiently in crop improvement approaches for diverse traits. According to the findings of this study, plant height and leaf length had the highest PCV estimates, followed by leaf width. Leaf length and 50% flowering in days determines the low GCV and PCV. Furthermore, direct selection based on panicle weight, test weight, and straw weight had a high and positive effect on grain yield per plant in both the rainy and summer seasons, indicating the true relationship between these characters and grain yield per plant, which aids indirect selection for these traits and thus improves grain yield per plant. The top five genotypes were therefore chosen using the pre-harvest and post-harvest attribute observation list. Based on the average performance of the best genotypes in terms of grain yield components in the agroclimatic conditions of Prayagraj, the best five genotypes were Kangni-1 (GS-14), Kangni-7 (GPF-7), Kangni-6 (GS-55), Kangni-5 (GS-389), and Kangni-4 (GS-368). As these findings are based on 2 years of data, biochemical testing of these genotypes validated their consistency.

## Data availability statement

The original contributions presented in the study are included in the article/supplementary material, further inquiries can be directed to the corresponding author.

## Ethics statement

All the genotypes were obtained from the NBPGR and ICRICIT for this project by the Directorate of Research at SHUATS.

## Author contributions

Conceptualization and methodology, DS and KL; Data curation, DS, KL, and SM; Investigation, DS; Writing—original draft, DS and KL; Writing—review and editing, DS, KL, SM, IB, RL, SE, RC, and RK. All authors have read and agreed the submitted manuscript.
